# The Prognostic Value of Retraction Clefts in Chinese Invasive Breast Cancer Patients

**DOI:** 10.3389/pore.2021.1609743

**Published:** 2021-04-21

**Authors:** Liangliang Huang, Yujie Li, Jun Du, Heng Li, Mengmeng Lu, Yuting Wang, Wenchao Zhou, Wei Wang, Haibo Wu

**Affiliations:** ^1^ Department of Pathology, The First Affiliated Hospital of USTC, Division of Life Sciences and Medicine, University of Science and Technology of China, Hefei, China; ^2^ Intelligent Pathology Institute, Division of Life Sciences and Medicine, University of Science and Technology of China, Hefei, China; ^3^ School of Life Sciences, Division of Life Sciences and Medicine, University of Science and Technology of China, Hefei, China

**Keywords:** prognosis, clinicopathological features, invasive breast carcinoma, retraction clefts, Chinese

## Abstract

Some studies reported the correlation between retraction clefts (RCs) and the clinicopathological features as well as prognosis in invasive breast carcinoma. However, limited number of investigations have been done and controversial results were reported. Larger population studies around the world might help to provide more accurate and comprehensive information. Thus, we examined the correlation between the extent of RCs and the clinicopathological features as well as the prognosis in 541 invasive breast carcinoma samples from Central China in this study. The statistical analyses were performed with the Pearson χ2 tests and univariate Cox proportional hazards regression assays. Compared with other studies, lower RCs occurrence rate (15.5%) was observed in Chinese breast cancer patients and opposite association between the presence of RCs and lymph nodes metastasis was identified, in which both progression free survival (PFS) and overall survival (OS) were improved with the presence of RCs in our study. Besides, despite some statistically significant associations between RCs and molecular subtypes, RCs and estrogen receptor status, the results were largely depending on the stratification methods. Generally, no convincing association was detected between the extent of RCs and the clinicopathological features or prognosis. In sum, the extent of RCs showed limited value as a prognostic predictor in invasive breast carcinoma patients from Central China.

## Introduction

Breast cancer is the most common malignant tumor with high heterogeneity amongst women [1]. Recently, retraction clefts (RCs) in breast neoplasms attracted pathologists’ special attention as they could be easily identified and classified in hematoxylin-eosin (H&E) stained sections under an optical microscope. In tumor sections, the cavity with no endothelial cell lining around tumor glands or nests was recognized as RC. The mechanisms underlying RC formation remains unclear. Some studies proposed that RCs were related to the loss of basal cells in breast carcinoma and prostate adenocarcinoma [2–5], while others suggested that RCs were caused by abnormal stroma around the tumor [6–8]. Besides, the lymphatic vessels, namely ‘pre-lymphatic channels’, may also contribute to RC formation [9, 10].

Clinically, some studies in certain types of basal cell carcinomas indicated diagnostic and prognostic significance of RCs [6–8, 11–15], whereas controversial reports suggested that RCs are artifactual spaces caused by improper processing during tissue fixation, paraffin embedding or cutting, with no association with prognosis [16, 17]. In breast cancer, Acs et al. made valuable contributions on the clinical value of RCs (they called “retraction artifact” at that time) [4–6, 9]. They performed investigations on resected specimens and core needle biopsy materials and showed that 55.7%–64.8% samples harbored RCs. Significant association was identified between extensive RCs and nodal metastasis in both types of their samples. Besides, the extent of RCs was significantly related to poor prognosis in their studies. Thus, they believe that extensive RCs was not a random artifactual phenomenon merely due to inadequate fixation and processing, but rather it represented true prelymphatic space which altered tumor-stromal interactions and contributes to lymphatic spread, tumor progression and poor prognosis [4–6, 9]. Their reports were using the samples from Moffitt Cancer Center in Florida state and University of Pennsylvania Medical Center in Pennsylvania state of America. A few more studies from University of Maryland Medical Center in the United States, Osaka National Hospital in Japan and the Fourth Hospital of Hebei Medical University in China also provided evidence to support this idea [10, 12, 15]. In contrast, except for the explanation of RCs as tissue shrinkage caused by handling and fixation [18–22], Kos and Leniček believed no association between lymphangiogenesis and RCs in the patients from University Hospital in Zagreb, Croatia [23]. Therefore, it is important to analyze RCs’ clinical value in more patients from different population in larger areas.

In this study, we investigated the extent of RCs in 541 invasive breast cancer specimens from a single-center located in Central China and explored their associations with clinicopathological features and prognosis.

## Materials and Methods

### Patients and Clinicopathological Data

In this study, 541 formalin-fixed paraffin-embedded (FFPE) samples were collected from the patients with no special type invasive breast carcinoma at the Department of Pathology, the First Affiliated Hospital of USTC, Division of Life Sciences and Medicine, University of Science and Technology of China from 2010 to 2017. All samples were taken by surgical excision before the patients receiving chemotherapy or radiotherapy. This study obtained written informed consent from all the patients and was approved by the Ethics Committee of the First Affiliated Hospital of USTC, Division of Life Sciences and Medicine, University of Science and Technology of China.

Surgically resected specimens were fixed in 10% neutral buffered formalin with the cold ischemia time not exceeding 30 min. Then, they were processed according to standard pathologic procedures. In general, after retrieving, the specimens were embedded in paraffin using Leica ASP300S Fully Enclosed Tissue Processor (Heidelberger, Leica Biosystems Nussloch GmbH) according to the protocol recommended by the manufacturer. 4 μm and 2 μm thick sections were cut and routinely stained with hematoxylin-eosin (H&E) and immunohistochemical assay.

Disease stage and tumor grade were recorded according to the American Joint Committee on Cancer (AJCC) TNM staging system of the 8th edition and WHO Classification of Tumors of the Breast (5th edition, 2019) respectively [24, 25]. The Nottingham Prognostic Index (NPI) predicts the prognosis of the breast cancer patients based on tumor size, tumor grade, and lymph node status [26]. Progression free survival (PFS) was defined as the time from surgery date until first recurrence, second primary tumor, last follow-up or death from any cause. Meanwhile, overall survival (OS) was defined as the time from surgery until the last follow-up or date of death. In this study, 320 patients were followed up with the median time of 35 months (range 5 to 97 months).

### Morphologic Evaluation of Retraction Clefts

RCs are similar to lymphatic or blood vessels, surrounding tumor glands or nests, but without lining endothelium. The extent of RCs was determined by evaluating the proportion of clefts that affected the tumor nests in the whole section. For example, tumors with clefts that affected approximately 10% of tumor nests were classified as 10% RCs. Two pathologists independently evaluated H&E sections to estimate the extent of RCs in invasive breast carcinoma and the average score was given to each sample. Lymphatic/vascular invasion and thermal damage to tissues were not considered to be RCs. Invasive micropapillary carcinomas and invasive carcinomas with micropapillary components were excluded as well. To further reduce the evaluation bias, lower than 5% RCs were counted as RC negative in this study. After the evaluation of RCs, the receiver-operating characteristic (ROC) curves were calculated based on the clinicopathological features and prognosis. The factor with the largest area under ROC curve (AUC) was employed to define the predictive cut-off value of the extent of RC which was used in the following analyses.

### Molecular Subtypes of Breast Cancer

According to the expression of ER, PR, HER2 and Ki-67, invasive breast carcinoma can be grouped into four molecular subtypes: Luminal A-like, Luminal B-like, Triple-negative, and HER2-enriched [27]. ER, PR, HER2 and Ki-67 were evaluated by immunohistochemistry (IHC). The criterion of hormone receptor positivity is that the positive staining of tumor nucleus is greater than or equal to 1%, while HER-2 positive means 3 + on immunohistochemical staining or in those reported as 2 +, amplification on fluorescence *in situ* hybridization according to the current WHO guidelines [28].

### Statistical Analysis

The ROC curve was generated by GraphPad Prism software v5.01 (GraphPad, San Diego, CA, United States), the corresponding AUC, 95% confidence interval (CI) and *p* value were calculated together with the sensitivity and specificity table. The Pearson χ^2^ test was used to compare the relations between the extent of RCs and clinicopathological features. The effect of various tumor variables on prognosis was researched by univariate analysis using the Cox proportional hazards model. The Kaplan-Meier survival curves were plotted using GraphPad Prism software v5.01 (GraphPad, San Diego, CA, United States), and the log-rank test was used to determine significant differences. *p* < 0.05 was considered statistically significant. SPSS software v19.0.0 (IBM, NY, United States) was used for statistical analysis.

## Results

**FIGURE 1:**
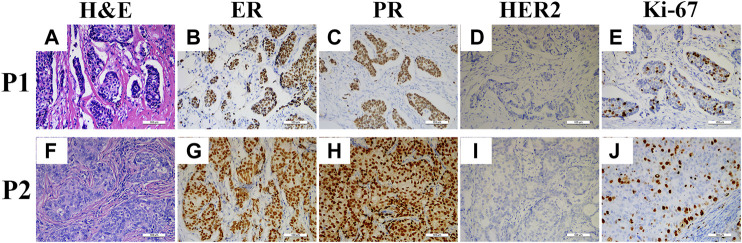
H&E and IHC staining sections of two patients. H&E staining of Patient 1 (P1) and Patient 2 (P2) showed retraction cleft positive **(A)** and negative **(B)**, respectively; ER positive in P1 **(B)** and P2 **(G)**; PR positive in P1 **(C)** and P2 **(H)**; HER2 negative in P1 **(D)** and P2 **(I)**; and Ki-67 labeling index <30% in P1 **(E)** and P2 **(J)**.

During the routine work, pathologists found RCs existed in the normal H&E stained sections (as shown in Figure 1A). To explore whether RCs play any pathological or prognostic value in breast cancer, the H&E sections of 541 breast cancer patients were carefully evaluated and the relationships between the RCs of these sections and the clinicopathological features as well as prognosis of the patients were analyzed in this study.

### Associations Between the Extent of RCs and Clinicopathological Features

Among the 541 breast cancer patients from Central China, RCs were detected in 84 cases (15.5%) (Table 1; Supplementary Table S2). The extent of RCs in these specimens ranged from 5% to 98% with median value of 60% (55.93% ± 23.31%; mean ± SD). Firstly, we determined the best cutoff value of the extent of RCs for our study. As shown in Supplementary Figure, the ROC curves were plotted under various clinicopathological characteristics and prognosis. The areas under the ROC curve (AUC) were between 0.5003−0.5217 (Supplementary Table S1), which showed no diagnostic discriminatory value. Even though, we set the cutoff point as 75% RCs (specificity = 96.83%, CI = 94.41%–98.22% and sensitivity = 8.287%, CI = 5.086%–13.22%) based on the largest AUC from HER2 assay group. Using this cutoff value, the association between RCs and clinicopathological features was analyzed. As shown in Table 1, specimens with >75% RCs were more common in HER2-enriched molecular subtype (*p* = 0.008), ER negative subgroup (*p* = 0.024) and HER2 positive subgroup (*p* = 0.024). In contrast, no statistically significant association was identified between the 75% RCs and patients’ age (*p* = 0.231), tumor stage (*p* = 0.659), lymph node status (*p* = 0.226), tumor size (*p* = 0.720), PR status (*p* = 0.366), or Ki67 index (*p* = 0.996).

**TABLE 1:** Association between the extent of retraction clefts and standard clinical, pathological and biological features of invasive breast carcinoma.

Parameter	Category	Total	Retraction clefts			Retraction clefts		
			Absence	Presence	*p*	≤75%	>75%	*p*
N		541	457 (84.5%)	84 (15.5%)	-	515 (95.2%)	26 (4.8%)	-
Age (years)	Median (IQR)	54 (43-56)	48 (42-56)	48 (44-56)	0.700	48 (43-56)	48 (42-52)	0.231
Tumor stages	I	13 (2.4%)	12 (3%)	1 (1%)	0.646	13 (2.5%)	0 (0%)	0.659
	II	206 (38.1%)	172 (38%)	34 (40%)		194 (37.7%)	12 (46.2%)	
	III	291 (53.8%)	248 (54%)	43 (51%)		279 (54.2%)	12 (46.2%)	
	Unknown	31 (5.7%)	25 (5%)	6 (7%)		29 (5.6%)	2 (7.7%)	
Tumor size	<2 cm	132 (24.4%)	115 (25%)	17 (20%)	0.127	128 (24.9%)	4 (15.4%)	0.720
	2-5 cm	349 (64.5%)	287 (63%)	62 (74%)		330 (64.1%)	19 (73.1%)	
	≥5 cm	58 (10.7%)	53 (12%)	5 (6%)		55 (10.7%)	3 (11.5%)	
	Unknown	2 (0.4%)	2(NA)	0(NA)		2 (0.4%)	0 (0%)	
Molecular subtypes	Luminal A	78 (14.4%)	65 (14%)	13 (15%)	**0.007**	77 (15.0%)	1 (3.8%)	**0.008**
	Luminal B	325 (60.1%)	273 (60%)	52 (62%)		310 (60.2%)	15 (57.7%)	
	HER2-enriched	70 (12.9%)	53 (12%)	17 (20%)		61 (11.8%)	9 (34.6%)	
	Triple negative	66 (12.2%)	64 (14%)	2 (2%)		65 (12.6%)	1 (3.8%)	
	Unknown	2 (0.4%)	2(NA)	0(NA)		2 (0.4%)	0 (0%)	
ER	Positive	389 (71.9%)	330 (72%)	59 (70%)	0.936	376 (73.0%)	13 (50.0%)	**0.024**
	Negative	146 (27.0%)	122 (27%)	24 (29%)		133 (25.8%)	13 (50.0%)	
	Unknown	6 (1.1%)	5 (1%)	1 (1%)		6 (1.2%)	0 (0%)	
PR	Positive	348 (64.3%)	304 (66%)	44 (52%)	0.189	334 (64.9%)	14 (53.8%)	0.366
	Negative	185 (34.2%)	146 (32%)	39 (46%)		173 (33.6%)	12 (46.2%)	
	Unknown	8 (1.5%)	7 (2%)	1 (1%)		8 (1.6%)	0 (0%)	
HER2	Positive	181 (33.5%)	148 (32%)	33 (39%)	0.252	166 (32.2%)	15 (57.7%)	**0.024**
	Negative	347 (64.1%)	297 (65%)	50 (60%)		336 (65.2%)	11 (42.3%)	
	Unknown	13 (2.4%)	12 (3%)	1 (1%)		13 (2.5%)	0 (0%)	
Ki67	≤30%	273 (50.5%)	230 (50%)	43 (51%)	0.924	260 (50.5%)	13 (50.0%)	0.996
	>30%	246 (45.5%)	208 (46%)	38 (45%)		234 (45.4%)	12 (46.2%)	
	Unknown	22 (4.1%)	19 (4%)	3 (4%)		21 (4.1%)	1 (3.8%)	
NPI	Good (2–3.4)	78 (14.4%)	67 (15%)	11 (13%)	0.218	74 (14.4%)	4 (15.4%)	0.218
	Moderate (3.4–5.4)	234 (43.3%)	190 (42%)	44 (52%)		222 (43.1%)	12 (46.2%)	
	Poor (>5.4)	174 (32.2%)	152 (33%)	22 (26%)		166 (32.2%)	8 (30.8%)	
	Unknown	55 (10.2%)	48 (11%)	7 (8%)		53 (10.3%)	2 (7.7%)	

Note: *p*<0.05 was considered statistically significant and those values are shown in bold. Abbreviations: IQR, interquartile range; NA, not available; ER, estrogen receptor; HER2, human epidermal growth factor receptor 2; PR, progesterone receptor; NPI, Nottingham Prognostic Index.

As all the AUC values were not clinically useful, we also examined the associations between RCs and clinicopathological features at different classification methods. Some significant relationships were identified in these assays. Such as the presence of RCs showed statistically significant association with molecular subtypes (*p* = 0.007), which probably was due to the rare RCs in triple-negative breast cancer (Table 1).

As shown in Supplementary Table S2 Assay I, significant associations were found between the extent of RCs and molecular subtypes (*p* = 0.024) and ER-status (*p* = 0.005). It seemed that ≤25% and 50%–75% RC groups had more luminal A-like samples, and >75% RC group had more HER2-enriched and ER-negative samples. However, no particular pattern was found in this assay. While the extents of RCs in tumor nests increased, the numbers of the luminal A-like, HER2-enriched and ER-negative samples fluctuated dramatically with no positive correlations. When stratifying the samples by 25% RCs in Assay II, significant correlation between RCs and molecular subtypes (*p* = 0.038) was detected (Supplementary Table S2). However, all the HER2-enriched samples were present in the >25% group, whereas ≤25% group had no HER2-enriched samples, which may be the main cause of the positive correlation. The same result was obtained in Assay Ⅲ as in Assay II, demonstrating that HER2-enriched subtype of breast cancers appeared only in tumors with a greater extent of RCs (>30%) (Supplementary Table S2). Similarly, tumors larger than 5 cm were exclusively located in the >25% and >30% groups in Assay II and Assay III, respectively. Interestingly, according to Assay III, it seemed to be a trend of increasing tumor size along with the increasing extent of RCs despite the lack of statistically significant difference between groups (*p* = 0.064). Besides, significant association between the extent of RCs (≤40% group vs. >40% group) and tumor sizes was identified in Assay IV, while ≥5 cm tumors only appeared in >40% RC group (Supplementary Table S2).

Taken together, despite statistical significances were identified in some minor points, no particular pattern of clinicopathological features was observed when classifying the extent of RCs with different settings. Therefore, no constant conclusion could be made based on the results of the above assays concerning the correlation between the extent of RCs and clinicopathological factors.

More directly, as shown in Figure 1, two patients showed similar clinicopathological characteristics (ER, PR, HER2 and Ki-67 staining) while they had entirely different extent of RCs. Therefore, we further examined all the samples and found that only 12% (8/68) of them had coincident RCs and clinicopathological phenotypes. However, even these 8 patients could be divided into 4 groups based on those phenotypes. Thus, these results demonstrated that no convincing data could support the pathological value of RCs in breast cancer.

### Relationship Between the Extent of RCs and Prognosis

NPI is widely used to predict the prognosis of breast cancer patients, which is divided into good (2–3.4), moderate (3.4–5.4) and poor (>5.4) classes [26]. As listed in Table 1, there was no significant association between the presence of RCs and prognosis. Similar results were obtained in the six assays of patient groups with different extent of RCs (Table 1 and Supplementary Table S2).

Furthermore, prognosis analysis was performed on the progression free survival (PFS) and overall survival (OS) time of 320 patients with a median follow-up period of 35 months. The Cox proportional hazards model and Kaplan-Meier survival curve were employed to evaluate the correlation between the extent of RCs and PFS or OS of breast cancer patients.

**FIGURE 2:**
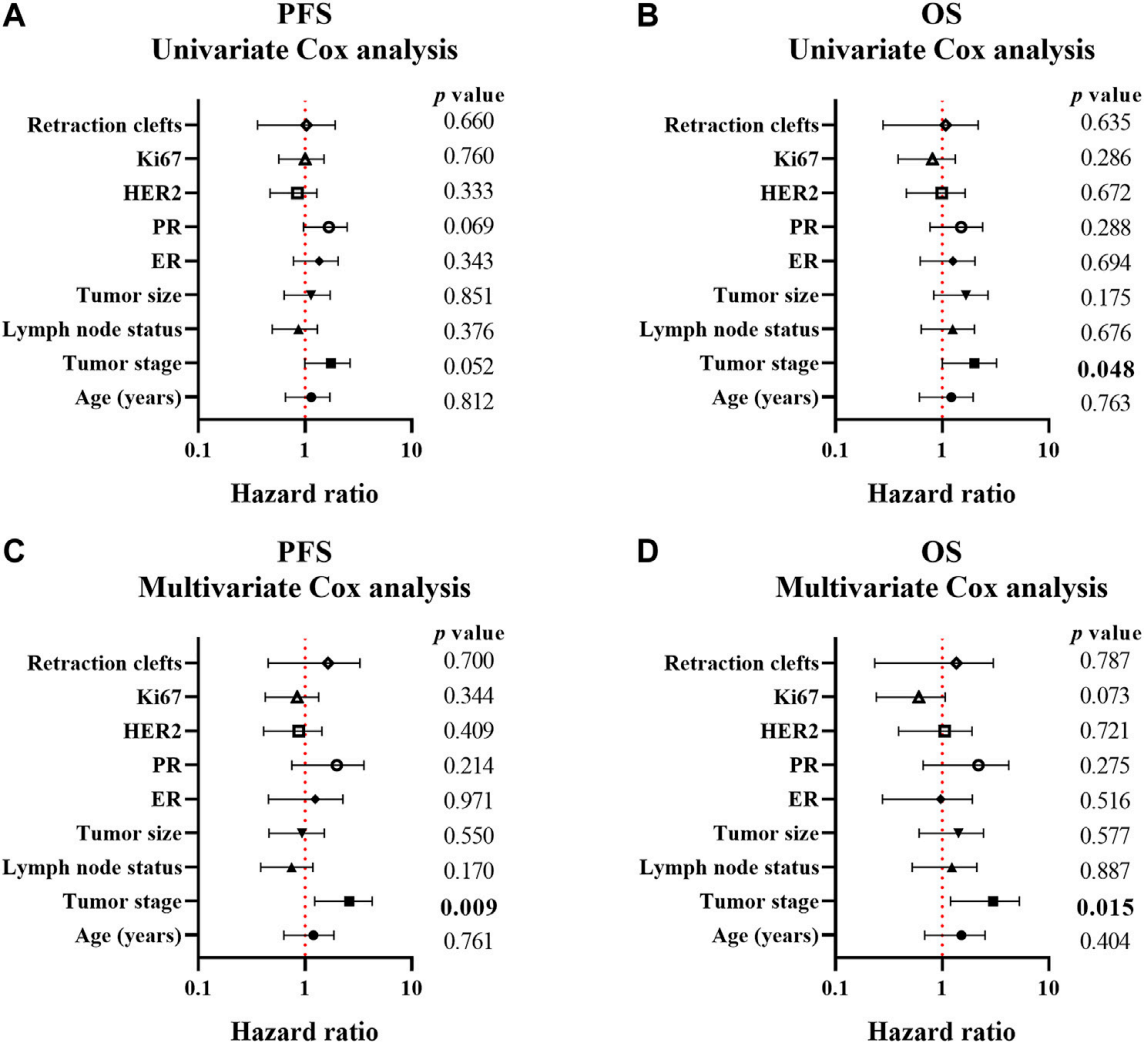
Forest plots of the association between clinicopathological parameters and progression-free/overall survival of breast cancer patients. A-B, forest plots of PFS **(A)** and OS **(B)** calculated by univariate Cox analysis; C-D, forest plots of PFS **(A)** and OS **(B)** calculated by multivariate Cox analysis. *p* < 0.05 was considered as statistically significant.

In univariate Cox regression analysis, tumor stage Ⅰ and Ⅱ (*p* = 0.048) correlated with a better OS. However, the presence of RCs showed no association with PFS (HR [95% CI] = 1.356 [0.792–2.322], *p* = 0.267) and OS (HR [95% CI] = 1.459 [0.769–2.766], *p* = 0.247) Supplementary Table S3 and Figures 2A,B). Considering that the most dramatic significances were identified in Assay IV when analyzing the association between the extent of RCs and standard clinical, pathological or biological features, the samples were stratified by 40% RCs and the univariate analysis was applied. However, no statistical significance was detected (Supplementary Table S3). In addition, the cutoff point of RCs (75%) was also analyzed, which indicated no significant relationship with PFS (HR [95% CI] = 0.828 [0.358-0.917], *p* = 0.660) and OS (HR [95% CI] = 0.779 [0.279–2.179], *p* = 0.635) as well (Supplementary Table S3 and Figures 2A,B).

Multivariate Cox analysis was also employed to assay the correlation between clinicopathological parameters and PFS/OS in this study. As shown in Supplementary Table S4, Figures 2C,D, only tumor stage correlated with both PFS (*p* = 0.009) and OS (*p* = 0.015) which indicated better prognosis in I and II tumor stage. However, 75% RCs was not a correlated factor for prognosis (PFS: HR [95% CI] = 1.215 [0.451–3.275], *p* = 0.700 and OS: HR [95% CI] = 0.838 [0.233–3.017], *p* = 0.787).

Considering the limited number of patients in >75% RCs group and the possible real effect of the RCs to prognosis under each clinicopathological factors, patients were divided into positive and negative groups according to clinicopathological parameters, and Cox analysis was performed to determine the correlations between prognosis and the presence of RCs or the extent of RCs (40% and 75% RCs). A significant difference in PFS was observed between lymph node positive and negative subgroups (*p* = 0.044), suggesting a correlation between prognosis and the presence of RCs (Supplementary Table S5). Meanwhile, worse PFS and OS was exhibited in >75% RCs group in the patients younger than 45 years when their tumors were diagnosed (*p* = 0.055 and 0.006, respectively) (Supplementary Table S5). Except these, no significance was identified in patients of different clinical, pathological and molecular subtypes.

**FIGURE 3:**
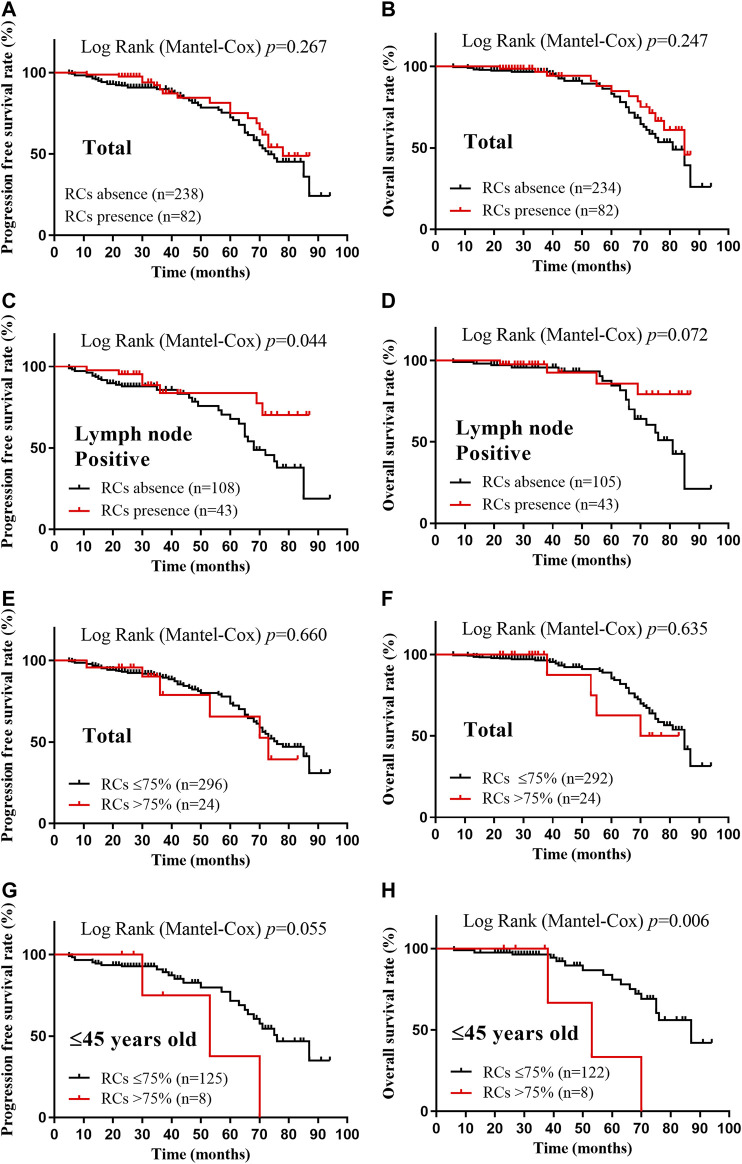
Kaplan-Meier survival curves according to the extent of retraction clefts for progression free survival and overall survival. A-B, PFS **(A)** and OS **(B)** rates of total patients with RCs presence; C-D, PFS **(C)** and OS **(D)** rates of lymph node positive patients with RCs presence; E-F, PFS **(E)** and OS **(F)** rates of total patients with >75% RCs; G-H, PFS **(G)** and OS **(H)** rates of ≤45 years old patients with >75% RCs. *p* < 0.05 was considered as statistically significant.

Using the Kaplan-Meier method and log-rank test, the PFS and OS were examined and no difference was found between the RC’s presence group and RC’s absence group (*p* = 0.267 and 0.247, respectively) (Figures 3A,B). Interestingly, the presence of RCs significantly improved the PFS of the patients with lymph nodes metastasis (*p* = 0.044), while it seemed to benefit the OS of these patients although without statistical significance (*p* = 0.072) (Figures 3C,D). When examined by 75% RCs, the younger patients (≤45 years old) with higher RCs (>75%) had worse PFS and OS (*p* = 0.055 and 0.006, respectively) (Figures 3G,H). Meanwhile, no significantly different PFS or OS was identified when separated the patients by 75% RCs (*p* = 0.660 and 0.635, respectively) (Figures 3E,F). Therefore, there was no convincing evidence that the extent of RCs could predict the prognosis of breast cancer patients.

## Discussion

In this study, we evaluated the clinicopathological and prognostic values of the extent of RCs in 541 invasive breast carcinoma samples. In general, RCs were present in 15.5% (84/541) of the samples in this study, which was much less than the report of Acs’ group [6] in 2015 as they identified variable extent of RCs in 55.7% of core needle biopsy and 64.8% of surgical materials of breast cancer, respectively. In the Japanese study [12], this ratio was 52.8% which was close to Acs’ results. Kos and Leniček even reported 92% occurrence ratio of RCs in their study based on Croatian [23]. Thus, we examined our sample selection and processing procedures from fixation to embedding, sectioning and staining, and compared the occurrence of RCs in the samples from different years (from 2010 to 2017) to identify the possible reason for our lower ratio of RCs. In general, the specimens were consecutive selected and nearly equally distributed in those 8 years. The tissue processing was nearly identical with little changes in the fixation time, embedding procedure and sectioning machines in our center during these years. No difference was identified in RCs occurrence during these years. Thus, the retraction clefts really existed in certain breast cancer specimens, even though their formation mechanism remains unclear. Then, we tried to address the issue about lower RCs occurrence ratio in our study. By comparison to the only research based on Chinese population (Deng’s study (483/2184, 22.1%) in 2018 from Hebei, China [15]), our result was also lower. Considering the presence of RCs in our study was defined as ≥5% retraction clefts in the whole section, the real occurrence ratio of RCs might be closer to the Deng’s report. Furthermore, the extensive RC (≥20%) was found in 10.9% (237/2184) cases in Deng’s study, which was even lower than our report (14.2%, 77/541). Thus, the occurrence ratio of RCs in Chinese patients was much lower than in other populations. This indicated that RCs might play different role among different races and more detailed investigations should be done in different regions all round the world.

The associations between the extent of RCs and clinicopathological characteristics were analyzed in this study. Except for the molecular subtypes, the presence of RCs showed no significant association with clinicopathological features. However, the small number of patients of triple-negative subtypes in RCs presence group limited the meaning of the association with regard to molecular subtypes. When patients were stratified into different groups by using different cut-off of RCs, some statistically significant correlations were found. For example, the extent of RCs correlated with HER2-enriched subtype in Assay I, II and III; with ER expression level in Assay I and III; and with tumor size in Assay III and IV. However, these results were confusing, and sometimes contradictory to each other. Therefore, no significant relationship between the extent of RCs and clinicopathological features was identified in our study.

The prognostic value of RCs was also assessed in this study. The association between the presence of RCs and prognosis was found in lymph node positive subgroup, in which both PFS and OS of the patients were improved with the presence of RCs. However, these results were opposite to previous investigations [4–6, 9, 15] reporting that the extensive RCs were linked to poor prognosis in lymph node positive invasive breast carcinoma. Meanwhile, Kos and Leniček found no relationship between RCs and lymph node metastases [23]. Therefore, the prognostic value of RCs was still of doubt, more researches on larger study populations are needed to get more certain conclusions.

In summary, we analyzed the relationships between the extent of RCs and different clinicopathological features or prognosis in 541 invasive breast carcinoma samples from Central China. Using seven assays based on different stratification methods of the extent of RCs as well as univariate and multivariate Cox regression analyses of subgroups, we found that the associations between RCs and clinicopathological features varied dramatically and were controversial among assays. Therefore, there may be no clear correlation between RCs and clinicopathological characteristics or prognosis in invasive breast carcinoma patients from Central China. We suggest that the RCs could provide little prognostic value for Chinese breast cancer patients.

## Data Availability

The original contributions presented in the study are included in the article/Supplementary Material, further inquiries can be directed to the corresponding authors.
