# Supplementary figures and images for: The Prognostic Value of Retraction Clefts in Chinese Invasive Breast Cancer Patients
Source: Pathol Oncol Res. 2021 Apr 21;27:1609743. doi: 10.3389/pore.2021.1609743 (PMC8262209; doi:10.3389/pore.2021.1609743)

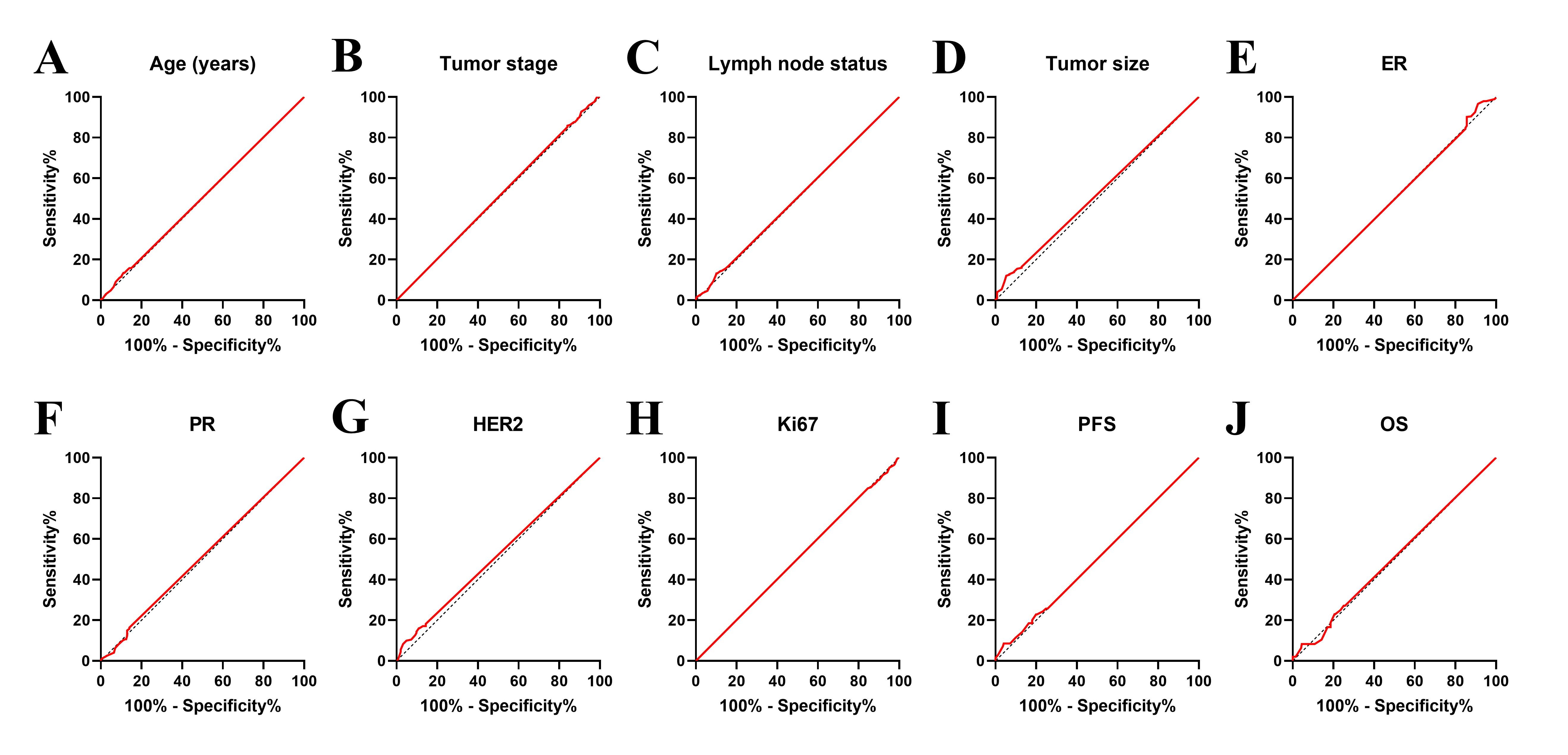

Supplement: Supplementary file 1 [file Image1.TIF]
