# Supplementary material for: The Prognostic Value of Retraction Clefts in Chinese Invasive Breast Cancer Patients
Source: Pathol Oncol Res. 2021 Apr 21;27:1609743. doi: 10.3389/pore.2021.1609743 (PMC8262209; doi:10.3389/pore.2021.1609743)
Supplement: Supplementary file 2 [file Table1.DOCX]

**Supplementary Table 1. Comparison of the area under the receiver-operator characteristic (ROC) curve (AUC) of the retraction clefts under various clinicopathological characteristics and prognosis**

| **Variables** | **AUC** | **95% CI** | ***p*** |
| --- | --- | --- | --- |
| **Age (years)**  **≤45 versus >45**    **Tumor stage**  **I and II versus III**    **Lymph node status**  **Positive versus negative**    **Tumor size**  **<2cm versus ≥2cm**    **ER**  **Positive versus negative**    **PR**  **Positive versus negative**    **HER2**  **Positive versus negative**    **Ki67**  **≤30% versus >30%**    **PFS**  **Progressive-free versus progressive**  **OS**  **Alive versus dead** | 0.5048  0.5064  0.5051  0.5211  0.5016  0.5091  0.5217  0.5003  0.5052  0.5054 | 0.4548-0.5549  0.4557-0.5571  0.4547-0.5555  0.4656-0.5765  0.4461-0.5571  0.4576-0.5606  0.4692-0.5742  0.4505-0.5501  0.4276-0.5827  0.4164-0.5943 | 0.8502  0.8042  0.8428  0.4670  0.9549  0.7287  0.4125  0.9892  0.8948  0.9058 |

Note: *p*<0.05 was considered statistically significant and those values are shown in bold.

Abbreviations: CI, confidence interval; PFS, progression free survival; OS, overall survival; ER, estrogen receptor; PR, progesterone receptor; HER2, human epidermal growth facto
