# Supplementary material for: The Prognostic Value of Retraction Clefts in Chinese Invasive Breast Cancer Patients
Source: Pathol Oncol Res. 2021 Apr 21;27:1609743. doi: 10.3389/pore.2021.1609743 (PMC8262209; doi:10.3389/pore.2021.1609743)
Supplement: Supplementary file 3 [file Table2.DOCX]

**Supplementary Table 2. Association between five different classified assays of the extent of retraction clefts and standard clinical, pathological and biological features of invasive breast carcinoma**

| **Parameter** | **Category** | **Total** | **Assay I** | | | | | **Assay II** | | | **Assay III** | | | **Assay IV** | | | **Assay V** | | |
| --- | --- | --- | --- | --- | --- | --- | --- | --- | --- | --- | --- | --- | --- | --- | --- | --- | --- | --- | --- |
|  |  |  | **≤25% RCs** | **25%-50% RCs** | **50%-75% RCs** | **>75% RCs** | ***p*** | **≤25% RCs** | **>25%RCs** | ***p*** | **≤30% RCs** | **>30%RCs** | ***p*** | **≤40% RCs** | **>40%RCs** | ***p*** | **≤50% RCs** | **>50%RCs** | ***p*** |
| **N**  **Age (years)**  **Tumor stages**  **Lymph node**  **status"**  **Tumor size**  **Molecular subtypes**  **ER**  **PR**  **HER2**  **Ki67**  **NPI** | **Median (IQR)**  **I**  **II**  **III**  **Unknown**  **0**  **1-3**  **>3**  **Unknown**  **<2cm**  **2-5cm**  **≥5cm**  **Unknown**  **Luminal A**  **Luminal B**  **HER2-enriched**  **Triple Negative**  **Unknown**  **Positive**  **Negative**  **Unknown**  **Positive**  **Negative**  **Unknown**  **Positive**  **Negative**  **Unknown**  **≤30%**  **>30%**  **Unknown**  **Good (2-3.4)**  **Moderate (3.4-5.4)**  **Poor (>5.4)**  **Unknown** | 541  54(43-56)  13  206  291  31  247  118  140  36  132  349  58  2  78  325  70  66  2  367  168  6  298  235  8  181  347  13  273  246  22  78  234  174  55 | 16(19.0%)  46.5(44-51)  1(6%)  7(44%)  7(44%)  1(6%)  9(56%)  4(25%)  2(13%)  1(6%)  5(31%)  11(69%)  0(NA)  0(NA)  5(31%)  10(63%)  0(NA)  1(6%)  0(NA)  14(88%)  2(13%)  0(NA)  11(69%)  5(31%)  0(NA)  4(25%)  12(75%)  0(NA)  10(63%)  6(38%)  0(NA)  4(25%)  8(50%)  3(19%)  1(6%) | 23(27.4%)  49(44-54.5)  0(NA)  7(30%)  15(65%)  1(4%)  9(39%)  6(26%)  7(30%)  1(4%)  6(26%)  16(70%)  1(4%)  0(NA)  1(4%)  17(74%)  5(22%)  0(NA)  0(NA)  17(74%)  6(26%)  0(NA)  12(52%)  11(48%)  0(NA)  10(43%)  13(57%)  0(NA)  11(48%)  11(48%)  1(4%)  2(9%)  13(57%)  6(26%)  2(9%) | 19(22.6%)  51(45-64.5)  0(NA)  8(42%)  9(47%)  2(11%)  7(37%)  6(32%)  6(32%)  0(NA)  2(11%)  16(84%)  1(5%)  0(NA)  6(32%)  10(53%)  3(16%)  0(NA)  0(NA)  16(84%)  2(11%)  1(5%)  12(63%)  6(32%)  1(5%)  4(21%)  14(74%)  1(5%)  9(47%)  9(47%)  1(5%)  1(5%)  11(58%)  5(26%)  2(11%) | 26(31.0%)  47(42-52)  0(NA)  12(46%)  12(46%)  2(8%)  14(54%)  3(12%)  9(35%)  0(NA)  4(15%)  19(73%)  3(12%)  0(NA)  1(4%)  15(58%)  9(35%)  1(4%)  0(NA)  12(46%)  14(54%)  0(NA)  9(35%)  17(65%)  0(NA)  15(58%)  11(42%)  0(NA)  13(50%)  12(46%)  1(4%)  4(15%)  12(46%)  8(31%)  2(8%) | -  0.290  0.400  0.501  0.499  **0.024**  **0.005**  0.091  0.060  0.865  0.681 | 16(19.0%)  46.5(44-51)  1(6%)  7(44%)  7(44%)  1(6%)  9(56%)  4(25%)  2(13%)  1(6%)  5(31%)  11(69%)  0(NA)  0(NA)  5(31%)  10(63%)  0(NA)  1(6%)  0(NA)  14(88%)  2(13%)  0(NA)  11(69%)  5(31%)  0(NA)  4(25%)  12(75%)  0(NA)  10(63%)  6(38%)  0(NA)  4(25%)  8(50%)  3(19%)  1(6%) | 68(81.0%)  49(45-56)  0(NA)  27(40%)  36(53%)  5(7%)  30(44%)  15(22%)  22(32%)  1(1%)  12(18%)  51(75%)  5(7%)  0(NA)  8(12%)  42(62%)  17(25%)  1(1%)  0(NA)  45(66%)  22(32%)  1(1%)  33(49%)  34(50%)  1(1%)  29(43%)  38(56%)  1(1%)  33(49%)  32(47%)  3(4%)  7(10%)  36(53%)  19(28%)  6(9%) | -  0.928  0.106  0.319  0.297  **0.038**  0.107  0.160  0.179  0.400  0.283 | 23(27.4%)  47(43.5-52)  1(4%)  10(43%)  11(48%)  1(4%)  13(57%)  4(17%)  5(22%)  1(4%)  8(35%)  15(65%)  0(NA)  0(NA)  5(22%)  17(74%)  0(NA)  1(4%)  0(NA)  21(91%)  2(9%)  0(NA)  14(61%)  9(39%)  0(NA)  7(30%)  16(70%)  0(NA)  13(57%)  9(39%)  1(4%)  6(26%)  11(48%)  5(22%)  1(4%) | 61(72.6%)  49(45-57)  0(NA)  24(39%)  32(52%)  5(8%)  26(43%)  15(25%)  19(31%)  1(2%)  9(15%)  47(77%)  5(8%)  0(NA)  8(13%)  35(57%)  17(28%)  1(2%)  0(NA)  38(62%)  22(36%)  1(2%)  30(49%)  30(49%)  1(2%)  26(43%)  34(56%)  1(2%)  30(49%)  29(48%)  2(3%)  5(8%)  33(54%)  17(28%)  6(10%) | -  0.905  0.257  0.449  0.064  **0.037**  **0.012**  0.375  0.283  0.508  0.118 | 28(33.3%)  48.5(44-54)  1(4%)  12(43%)  14(50%)  1(4%)  15(54%)  6(21%)  6(21%)  1(4%)  10(36%)  18(64%)  0(NA)  0(NA)  6(21%)  19(68%)  2(7%)  1(4%)  0(NA)  23(82%)  5(18%)  0(NA)  16(57%)  12(43%)  0(NA)  10(36%)  18(64%)  0(NA)  16(57%)  11(39%)  1(4%)  6(21%)  16(57%)  5(18%)  1(4%) | 56(66.7%)  49(45-58)  0(NA)  22(39%)  29(52%)  5(9%)  24(43%)  13(23%)  18(32%)  1(2%)  7(13%)  44(79%)  5(9%)  0(NA)  7(13%)  33(59%)  15(27%)  1(2%)  0(NA)  36(64%)  19(34%)  1(2%)  28(50%)  27(48%)  1(2%)  23(41%)  32(57%)  1(2%)  27(48%)  27(48%)  2(4%)  5(9%)  28(50%)  17(30%)  6(11%) | -  0.916  0.372  0.538  **0.018**  0.171  0.113  0.591  0.591  0.431  0.189 | 39(46.4%)  48(43.5-54)  1(3%)  14(36%)  22(56%)  2(5%)  18(46%)  10(26%)  9(23%)  2(5%)  11(28%)  27(69%)  1(3%)  0(NA)  6(15%)  27(69%)  5(13%)  1(3%)  0(NA)  31(79%)  8(21%)  0(NA)  23(59%)  16(41%)  0(NA)  14(36%)  25(64%)  0(NA)  21(54%)  17(44%)  1(3%)  6(15%)  21(54%)  9(23%)  3(8%) | 45(53.6%)  49(45-59)  0(NA)  20(44%)  21(47%)  4(9%)  21(47%)  9(20%)  15(33%)  0(NA)  6(13%)  35(78%)  4(9%)  0(NA)  7(16%)  25(56%)  12(27%)  1(2%)  0(NA)  28(62%)  16(36%)  1(2%)  21(47%)  23(51%)  1(2%)  19(42%)  25(56%)  1(2%)  22(49%)  21(47%)  2(4%)  5(11%)  23(51%)  13(29%)  4(9%) | -  0.676  0.390  0.603  0.143  0.454  0.112  0.306  0.499  0.712  0.746 |

Note: *p*<0.05 was considered statistically significant and those values are shown in bold.

Abbreviations: RCs, retraction clefts; IQR, interquartile range; NA, not available; ER, estrogen receptor; HER2, human epidermal growth factor receptor 2; PR, progesterone receptor; NPI, Nottingham Prognostic Index
