# Supplementary material for: The Prognostic Value of Retraction Clefts in Chinese Invasive Breast Cancer Patients
Source: Pathol Oncol Res. 2021 Apr 21;27:1609743. doi: 10.3389/pore.2021.1609743 (PMC8262209; doi:10.3389/pore.2021.1609743)
Supplement: Supplementary file 4 [file Table3.DOCX]

**Supplementary Table 3. Univariate Cox analysis of the correlation between clinicopathological parameters and progression-free/overall survival of breast cancer patients**

| **Variables** | **PFS** | | **OS** | |
| --- | --- | --- | --- | --- |
|  | **HR (95% CI)** | ***p*** | **HR (95% CI)** | ***p*** |
| **Age (years)**  **≤45 versus >45**    **Tumor stage**  **I and II versus III**    **Lymph node status**  **Positive versus negative**    **Tumor size**  **<2cm versus ≥2cm**    **Molecular subtypes**  **Luminal and Triple negative versus HER2-enriched**  **Luminal and HER2-enriched versus Triple negative**  **Luminal versus Triple negative and HER2-enriched**    **ER**  **Positive versus negative**    **PR**  **Positive versus negative**    **HER2**  **Positive versus negative**    **Ki67**  **≤30% versus >30%**    **Retraction clefts**  **≤75% versus >75%**  **≤40% versus >40%**  **Presence versus absence** | 1.060(0.656-1.712)  1.623(0.997-2.644)  0.803(0.493-1.306)  1.049(0.638-1.725)  1.508(0.850-2.677)  0.932(0.475-1.831)  0.727(0.445-1.188)  1.263(0.779-2.048)  1.552(0.967-2.491)  0.780(0.471-1.291)  0.927(0.569-1.508)  0.828(0.358-1.917)  1.330(0.726-2.437)  1.356(0.792-2.322) | 0.812  0.052  0.376  0.851  0.160  0.839  0.203  0.343  0.069  0.333  0.760  0.660  0.356  0.267 | 1.093(0.612-1.953)  1.802(1.005-3.233)  1.130(0.637-2.004)  1.499(0.835-2.692)  1.367(0.658-2.841)  1.209(0.563-2.598)  1.362(0.753-2.465)  1.126(0.622-2.038)  1.361(0.771-2.402)  0.872(0.463-1.643)  0.715(0.386-1.325)  0.779(0.279-2.179)  1.650(0.771-3.534)  1.459(0.769-2.766) | 0.763  **0.048**  0.676  0.175  0.402  0.626  0.307  0.694  0.288  0.672  0.286  0.635  0.197  0.247 |

Note: *p*<0.05 was considered statistically significant and those values are shown in bold.

Abbreviations: HR, hazard ratio; CI, confidence interval; PFS, progression free survival; OS, overall survival; ER, estrogen receptor; PR, progesterone receptor; HER2, human epidermal growth factor; RCs, retraction clefts
