# Supplementary material for: The Prognostic Value of Retraction Clefts in Chinese Invasive Breast Cancer Patients
Source: Pathol Oncol Res. 2021 Apr 21;27:1609743. doi: 10.3389/pore.2021.1609743 (PMC8262209; doi:10.3389/pore.2021.1609743)
Supplement: Supplementary file 5 [file Table4.DOCX]

**Supplementary Table 4. Multivariate Cox analysis of the correlation between clinicopathological parameters and progression-free/overall survival of breast cancer patients**

| **Variables** | **PFS** | | **OS** | |
| --- | --- | --- | --- | --- |
|  | **HR (95% CI)** | ***p*** | **HR (95% CI)** | ***p*** |
| **Age (years)**  **≤45 versus >45**    **Tumor stage**  **I and II versus III**    **Lymph node status**  **Positive versus negative**    **Tumor size**  **<2cm versus ≥2cm**    **ER**  **Positive versus negative**    **PR**  **Positive versus negative**    **HER2**  **Positive versus negative**    **Ki67**  **≤30% versus >30%**    **Retraction clefts**  **≤75% versus >75%** | 1.087(0.634-1.864)  2.292(1.233-4.258)  0.674(0.384-1.184)  0.834(0.459-1.514)  1.015(0.455-2.265)  1.639(0.752-3.569)  0.767(0.409-1.439)  0.757(0.425-1.347)  1.215(0.451-3.275) | 0.761  **0.009**  0.170  0.550  0.971  0.214  0.409  0.344  0.700 | 1.320(0.688-2.532)  2,519(1.196-5.309)  1.052(0.523-2.117)  1.218(0.609-2.437)  0.725(0.275-1.914)  1.675(0.664-4.226)  0.865(0.392-1.913)  0.507(0.241-1.066)  0.838(0.233-3.017) | 0.404  **0.015**  0.887  0.577  0.516  0.275  0.721  0.073  0.787 |

Note: *p*<0.05 was considered statistically significant and those values are shown in bold.

Abbreviations: HR, hazard ratio; CI, confidence interval; PFS, progression free survival; OS, overall survival; ER, estrogen receptor; PR, progesterone receptor; HER2, human epidermal growth factor; RCs, retraction clefts
