# Supplementary material for: The Prognostic Value of Retraction Clefts in Chinese Invasive Breast Cancer Patients
Source: Pathol Oncol Res. 2021 Apr 21;27:1609743. doi: 10.3389/pore.2021.1609743 (PMC8262209; doi:10.3389/pore.2021.1609743)
Supplement: Supplementary file 6 [file Table5.DOCX]

**Supplementary Table 5. Univariate Cox analysis of the correlation between the extent of retraction clefts and progression-free/overall survival according to different clinicopathological parameters**

| **Variables** | **PFS** | | **OS** | |
| --- | --- | --- | --- | --- |
|  | **HR (95% CI)** | ***p*** | **HR (95% CI)** | ***p*** |
| **≤45 years old**  **Presence versus absence**  **≤40% versus >40%**  **≤75% versus >75%**    **>45 years old**  **Presence versus absence**  **≤40% versus >40%**  **≤75% versus >75%**    **Tumor stage I and II**  **Presence versus absence**  **≤40% versus >40%**  **≤75% versus >75%**    **Tumor stage III**  **Presence versus absence**  **≤40% versus >40%**  **≤75% versus >75%**    **Lymph node Negative**  **Presence versus absence**  **≤40% versus >40%**  **≤75% versus >75%**  **Lymph node Positive**  **Presence versus absence**  **≤40% versus >40%**  **≤75% versus >75%**    **Tumor size <2cm**  **Presence versus absence**  **≤40% versus >40%**  **≤75% versus >75%**    **Tumor size≥2cm**  **Presence versus absence**  **≤40% versus >40%**  **≤75% versus >75%**    **Luminal and Triple negative subtypes**  **Presence versus absence**  **≤40% versus >40%**  **≤75% versus >75%**    **HER2-enriched subtype**  **Presence versus absence**  **≤40% versus >40%**  **≤75% versus >75%**  **Luminal and HER2-enriched subtypes**  **Presence versus absence**  **≤40% versus >40%**  **≤75% versus >75%**  **Triple negative subtype**  **Presence versus absence**  **≤40% versus >40%**  **≤75% versus >75%**  **Luminal subtypes**  **Presence versus absence**  **≤40% versus >40%**  **≤75% versus >75%**    **HER2-enriched and Triple negative subtypes**  **Presence versus absence**  **≤40% versus >40%**  **≤75% versus >75%** | 1.413(0.576-3.463)  1.010(0.385-2.645)  0.295(0.085-1.025)  1.381(0.695-2.741)  1.585(0.725-3.468)  2.123(0.504-8.934)  1.153(0.496-2.681)  0.921(0.376-2.255)  0.846(0.199-3.603)  1.179(0.563-2.471)  1.392(0.575-3.372)  0.951(0.286-3.159)  0.711(0.329-1.539)  0.931(0.374-2.320)  0.820(0.242-2.776)  2.329(1.022-5.304)  1.637(0.719-3.726)  1.080(0.257-4.543)  0.785(0.290-2.214)  1.070(0.317-3.610)  -  1.539(0.806-2.939)  1.347(0.665-2.728)  0.942(0.371-2.396)  1.254(0.683-2.304)  1.344(0.656-2.753)  0.768(0.239-2.469)  1.845(0.582-5.852)  1.558(0.489-4.963)  1.039(0.291-3.711)  1.390(0.797-2.424)  1.382(0.744-2.567)  0.834(0.358-1.943)  -  -  -  1.263(0.670-2.382)  1.393(0.668-2.906)  0.745(0.230-2.412)  1.511(0.516-4.428)  1.245(0.425-3.648)  0.908(0.270-3.048) | 0.450  0.985  0.055  0.357  0.249  0.305  0.740  0.857  0.821  0.662  0.463  0.935  0.387  0.879  0.750  **0.044**  0.240  0.916  0.633  0.913  -  0.192  0.407  0.901  0.466  0.419  0.657  0.299  0.453  0.953  0.246  0.306  0.675  -  -  -  0.471  0.377  0.623  0.451  0.690  0.875 | 1.757(0.590-5.236)  1.434(0.421-4.882)  0.159(0.043-0.593)  1.456(0.640-3.315)  1.813(0.683-4.811)  -  1.454(0.491-4.305)  1.424(0.420-4.827)  -  1.170(0.494-2.773)  1.518(0.515-4.472)  0.450(0.128-1.591)  0.859(0.365-2.024)  1.201(0.401-3.593)  0.838(0.190-3.697)  2.699(0.913-7.976)  1.956(0.665-5.757)  1.119(0.147-8.497)  0.708(0.231-2.169)  1.280(0.293-5.588)  -  1.695(0.765-3.756)  1.623(0.658-4.001)  0.748(0.222-2.515)  1.457(0.708-3.000)  1.945(0.759-4.983)  0.686(0.164-2.871)  1.596(0.395-6.444)  -  1.059(0.218-5.136)  1.486(0.762-2.894)  1.709(0.785-3.720)  0.759(0.269-2.143)  -  -  -  1.465(0.686-3.128)  2.017(0.773-5.263)  0.619(0.146-2.623)  1.359(0.386-4.786)  1.148(0.326-4.038)  0.955(0.217-4.213) | 0.311  0.564  **0.006**  0.371  0.232  -  0.499  0.570  -  0.722  0.449  0.215  0.729  0.744  0.816  0.072  0.223  0.914  0.545  0.743  -  0.193  0.293  0.639  0.307  0.166  0.606  0.512  -  0.944  0.245  0.177  0.603  -  -  -  0.324  0.152  0.515  0.633  0.830  0.952 |

Continued

| **Variables** | **PFS** | | **OS** | |
| --- | --- | --- | --- | --- |
|  | **HR (95% CI)** | ***p*** | **HR (95% CI)** | ***p*** |
| **ER Negative**  **Presence versus absence**  **≤40% versus >40%**  **≤75% versus >75%**    **ER Positive**  **Presence versus absence**  **≤40% versus >40%**  **≤75% versus >75%**  **PR Negative**  **Presence versus absence**  **≤40% versus >40%**  **≤75% versus >75%**    **PR Positive**  **Presence versus absence**  **≤40% versus >40%**  **≤75% versus >75%**    **HER2 Negative**  **Presence versus absence**  **≤40% versus >40%**  **≤75% versus >75%**  **HER2 Positive**  **Presence versus absence**  **≤40% versus >40%**  **≤75% versus >75%**    **Ki67 labelling index ≤30%**  **Presence versus absence**  **≤40% versus >40%**  **≤75% versus >75%**  **Ki67 labelling index >30%**  **Presence versus absence**  **≤40% versus >40%**  **≤75% versus >75%** | 1.466(0.551-3.900)  1.102(0.414-2.933)  0.959(0.273-3.376)  1.174(0.611-2.255)  1.390(0.642-3.007)  0.669(0.160-2.798)  1.318(0.623-2.787)  1.298(0.542-3.108)  0.891(0.262-3.038)  1.313(0.602-2.865)  1.309(0.561-3.057)  1.025(0.244-4.305)  1.255(0.649-2.428)  1.222(0.588-2.539)  0.969(0.232-4.055)  1.390(0.548-3.522)  1.415(0.481-4.165)  0.870(0.248-3.044)  1.365(0.597-3.123)  1.197(0.479-2.988)  0.943(0.212-4.190)  1.249(0.587-2.657)  1.173(0.513-2.679)  0.755(0.229-2.485) | 0.443  0.845  0.948  0.631  0.403  0.582  0.471  0.558  0.854  0.493  0.533  0.973  0.500  0.591  0.966  0.488  0.528  0.827  0.461  0.700  0.943  0.564  0.706  0.644 | 1.633(0.464-5.747)  1.235(0.351-4.347)  0.962(0.202-4.596)  1.331(0.623-2.843)  1.854(0.711-4.836)  0.655(0.087-4.904)  1.154(0.482-2.766)  1.157(0.394-3.403)  0.882(0.195-3.994)  1.795(0.704-4.578)  2.083(0.706-6.142)  1.057(0.140-7.961)  1.504(0.679-3.329)  1.870(0.722-4.844)  1.240(0.167-9.208)  1.416(0.430-4.660)  0.992(0.272-3.619)  0.557(0.111-2.805)  1.867(0.587-5.940)  2.841(0.633-12.750)  -  1.310(0.564-3.045)  1.062(0.428-2.640)  0.439(0.130-1.481) | 0.445  0.742  0.962  0.461  0.207  0.680  0.748  0.791  0.870  0.221  0.184  0.957  0.314  0.197  0.834  0.567  0.991  0.478  0.291  0.173  -  0.530  0.896  0.185 |

Note: *p*<0.05 was considered statistically significant and those values are shown in bold.

Abbreviations: HR, hazard ratio; CI, confidence interval; PFS, progression free survival; OS, overall survival; ER, estrogen receptor; PR, progesterone receptor; HER2, human epidermal growth factor; RCs, retraction clefts
